# Structural and Evolutionary Analyses of PR-4 SUGARWINs Points to a Different Pattern of Protein Function

**DOI:** 10.3389/fpls.2021.734248

**Published:** 2021-09-09

**Authors:** Lorhenn Bryanda Lemes Maia, Humberto D’Muniz Pereira, Richard Charles Garratt, José Brandão-Neto, Flavio Henrique-Silva, Danyelle Toyama, Renata O. Dias, José Fernando Ruggiero Bachega, Julia Vasconcellos Peixoto, Marcio C. Silva-Filho

**Affiliations:** ^1^Programa de Pós-Graduação em Biotecnologia, Universidade Católica Dom Bosco, Campo Grande, Brazil; ^2^Instituto de Física de São Carlos, Universidade de São Paulo, São Carlos, Brazil; ^3^Diamond Light Source, Harwell Science and Innovation Campus Didcot, Harwell, United Kingdom; ^4^Departamento de Genética e Evolução, Universidade Federal de São Carlos, São Carlos, Brazi; ^5^Instituto de Ciências Biológicas, Universidade Federal de Goiás, Goiânia, Brazil; ^6^Departamento de Farmacociências, Universidade Federal de Ciências da Saúde de Porto Alegre, Porto Alegre, Brazil; ^7^Programa de Pós-Graduação de Biologia Celular e Molecular, Universidade Federal do Rio Grande do Sul, Porto Alegre, Brazil; ^8^Departamento de Genética, Escola Superior de Agricultura Luiz de Queiroz, Universidade de São Paulo, Piracicaba, Brazil

**Keywords:** SUGARWIN, BARWIN, crystallography, flexible loop, PR-4, phylogenetic analysis

## Abstract

SUGARWINs are PR-4 proteins associated with sugarcane defense against phytopathogens. Their expression is induced in response to damage by *Diatraea saccharalis* larvae. These proteins play an important role in plant defense, in particular against fungal pathogens, such as *Colletothricum falcatum* (Went) and *Fusarium verticillioides*. The pathogenesis-related protein-4 (PR-4) family is a group of proteins equipped with a BARWIN domain, which may be associated with a chitin-binding domain also known as the hevein-like domain. Several PR-4 proteins exhibit both chitinase and RNase activity, with the latter being associated with the presence of two histidine residues H11 and H113 (BARWIN) [H44 and H146, SUGARWINs] in the BARWIN-like domain. In sugarcane, similar to other PR-4 proteins, SUGARWIN1 exhibits ribonuclease, chitosanase and chitinase activities, whereas SUGARWIN2 only exhibits chitosanase activity. In order to decipher the structural determinants involved in this diverse range of enzyme specificities, we determined the 3-D structure of SUGARWIN2, at 1.55Å by X-ray diffraction. This is the first structure of a PR-4 protein where the first histidine has been replaced by asparagine and was subsequently used to build a homology model for SUGARWIN1. Molecular dynamics simulations of both proteins revealed the presence of a flexible loop only in SUGARWIN1 and we postulate that this, together with the presence of the catalytic histidine at position 42, renders it competent as a ribonuclease. The more electropositive surface potential of SUGARWIN1 would also be expected to favor complex formation with RNA. A phylogenetic analysis of PR-4 proteins obtained from 106 Embryophyta genomes showed that both catalytic histidines are widespread among them with few replacements in these amino acid positions during the gene family evolutionary history. We observe that the H11 replacement by N11 is also present in two other sugarcane PR-4 proteins: SUGARWIN3 and SUGARWIN4. We propose that RNase activity was present in the first Embryophyta PR-4 proteins but was recently lost in members of this family during the course of evolution.

## Introduction

Plant responses to insects and pathogens are complex and modulate the expression of a large number of genes, many of which are believed to have a direct role in plant defense (Xu et al., 1994; Agrawal et al., 2003; Banno et al., 2005; Franco et al., 2017). Pathogen recognition by plants activates the host defense response resulting in the accumulation of pathogenesis-related proteins (PR proteins) (Pieterse and van Loon, 1999). Several genes that encode wound-inducible proteins (WIN) have been identified in various plant species (Ryan, 1990; Christopher et al., 2004; Schilmiller and Howe, 2005; Medeiros et al., 2012).

The pathogenesis-related protein-4 (PR-4) family is a group of proteins composed of a BARWIN domain, which was first identified in the BARWIN protein from barley (Hejgaard et al., 1992; Svensson et al., 1992). Although the BARWIN domain can be found in association with a chitin-binding domain typical of lectins, known as a hevein-like domain (Broekaert et al., 1990), several proteins show the absence of this protein motif. PR-4 proteins composed only by BARWIN domains were identified in several plants, such as: tobacco (Friedrich et al., 1991), tomato (Linthorst et al., 1991), *Arabidopsis* (Potter et al., 1993), wheat (Caruso et al., 1999), *W. japonica* (Kiba et al., 2003), maize (Bravo et al., 2003), rice (Agrawal et al., 2003; Zhu et al., 2006) and *L. radiata* (Li et al., 2010). The PR-4 proteins are grouped into class I and class II based on the presence or absence of this chitin-binding domain (Neuhaus et al., 1996).

The PR-4 BARWIN homologs of several plant species have been associated with the plant responses to fungal infection and mechanical wounding (Linthorst et al., 1991; Hejgaard et al., 1992; Caruso et al., 1999, 2001; Agrawal et al., 2003; Bravo et al., 2003; Kiba et al., 2003; Zhu et al., 2006; Li et al., 2010; Medeiros et al., 2012; Bai et al., 2013; Franco et al., 2014, 2019; Menezes et al., 2014; Souza et al., 2017). PR-4 proteins have been classified as chitinases (Neuhaus et al., 1996; Van Loon and Van Strien, 1999); however, several studies have also reported RNase activity for BARWIN-like proteins (Bertini et al., 2009, 2012; Guevara-Morato et al., 2010; Bai et al., 2013; Huet et al., 2013; Franco et al., 2014; Menezes et al., 2014; Kim and Hwang, 2015). RNA-binding sites have been described for WHEATWIN1 (Bai et al., 2013) and CARWIN (Huet et al., 2013), showing two important histidine residues necessary for RNase activity, one at position 11 and another at position 113, numbered according to the mature BARWIN sequence, which may be used as the reference residue numbering in this text (Bai et al., 2013). Combined DNase and RNase antifungal activities were also observed for the *Capsicum chinense* PR-4 protein (Guevara-Morato et al., 2010) and the *Theobroma cacao* TcPR-4b protein (Menezes et al., 2014).

Our previous studies have identified two homologous genes to BARWIN in sugarcane: *SUGARWIN1* and *SUGARWIN2* (Falco et al., 2001; Medeiros et al., 2012). SUGARWIN proteins (sugarcane wound-inducible proteins) are believed to be part of a defense mechanism against pathogenic fungi in sugarcane plants *via* a salicylic acid (SA)-independent and jasmonic acid (JA)- dependent pathway. These proteins are secreted to the apoplasts, are strongly upregulated in response to mechanical wounding, *Diatraea saccharalis* (Fabricius) attack, methyl jasmonate treatment and to a lesser extent to sugarcane fungus (Medeiros et al., 2012). Despite their high expression levels in response to *D. saccharalis* attack, these proteins have no effect on insect development and mortality. However, SUGARWINs have antimicrobial effects in pathogenic fungus causing changes in hyphae morphology and leading to cell death by apoptosis (Medeiros et al., 2012; Franco et al., 2014). We have recently shown that *Fusarium verticillioides*, a pathogenic fungus, modulates sugarcane plants and *Diatrea saccharalis* moths and larvae to increase its dissemination in the field (Franco et al., 2021). The antifungal activity of SUGARWIN proteins is likely due to its multiple enzyme properties, such as: chitosanase, RNase and chitinase activities. This variable enzyme specificity has been suggested to be a result of a divergent amino acid composition at the substrate- binding site of these two proteins (Franco et al., 2014).

In order to understand how these SUGARWINs acquired these divergent activities, here we analyze their evolutionary history and their three-dimensional structures, looking for a correlation between structural and functional properties. Furthermore, we also describe two new putative SUGARWIN genes: *SUGARWIN3* and *SUGARWIN4*, which may also lack the canonical RNase activity observed in other PR-4 proteins as both have asparagine instead of histidine in the amino acid position 11 (43—SUGARWIN3; 38—SUGARWIN4).

## Materials and Methods

### Recombinant Expression and Purification of SUGARWIN2

The heterologous expression of SUGARWIN2 was performed in *Pichia pastoris* as previously described (Medeiros et al., 2012). Basically, a single colony of *P. pastoris* containing the pPICZα-SUG2, that contains the coding region for SUGARWIN2 was inoculated in 10 ml of BMGY medium [1% yeast extract, 2% peptone, 100 mM potassium phosphate buffer (pH 7.0), 1.34% YNB, 10^–5^% biotin, and 1% glycerol], which was incubated at 30°C until reaching an optical density (OD) at 600 nm of about 5. This culture was used to inoculate 500 ml of BMGY and was grown to an OD of 4–5. The cells were harvested by centrifugation at 1,500 × *g* for 5 min, resuspended in 100 ml of BMGY with 0.5% methanol instead of glycerol, and incubated at 28°C. For induction, methanol was added to each sample every 24 h to maintain a final concentration of 0.75%. After 96 h, the cells were harvested by centrifugation at 1,500 × *g* for 5 min, and the supernatant was filtered with a 0.45 mm membrane (Millipore, Bedford, MA, United States). The recombinant protein in the supernatant was purified by immobilized metal affinity chromatography (IMAC) using Ni-NTA-agarose (Qiagen) pre-equilibrated with purification buffer (10 mM Tris–HCl, pH 8.0; 50 mM NaH2PO4; and 100 mM NaCl). After binding, the proteins were eluted with two-column volumes of purification buffer containing increasing imidazole concentrations (10, 25, 50, 75, 100, and 250 mM). The fractions containing the recombinant protein were pooled, dialyzed in phosphate-buffered saline (PBS, pH 7.4) (137 mM NaCl, 2.7 mM KCl, 10 mM Na2PO4 and 2 mM KH2PO4) and sterilized with a 0.22 mm filter (Millipore). The protein concentration was determined using a BCA protein assay kit (Pierce).

The purification of the protein was monitored using 12% SDS-PAGE (Supplementary Figure 1). The recombinant SUGARWIN2 sample was further purified in an AKTA Purifier (GE, Healthcare) with a gel filtration column (Superdex 75) in TRIS solution (20 mM; pH 7.4), with 200 mM of NaCl. The final purification of the recombinant protein SUGARWIN2 was monitored by Dynamic Light Scattering (DLS) (Supplementary Figure 2).

### Crystallization, Data Collection, Structure Determination and Refinement of SUGARWIN2

Recombinant SUGARWIN2 from gel filtration experiment at 7 mg/mL were crystallized by the sitting drop vapor diffusion method using the Morpheus screening kit (Molecular Dimensions). 200 nL drops were setup employing a Crystal Gryphon robot (Art Robbins). The plate was incubated at 18°C. Several conditions resulted in bipyramidal crystals after 1 week. The crystals were flash-cooled in liquid nitrogen for data collection. The structure presented here is from a crystal grown in 0.1 M MES/imidazole pH 6.5, 10% w/v PEG 4000, 20% v/v glycerol 0.03 M sodium fluoride 0.03 M sodium bromide and 0.3 M sodium iodide.

X-ray diffraction data were collected on beamline I24 of the Diamond Light Source (DLS), United Kingdom, (λ = 0.96863 Å) using a PILATUS3 6 M detector. The diffraction data were indexed, integrated and scaled using the xia2 package (REF). The data were processed up to 1.55 Å resolution. The structure was solved by molecular replacement using Phaser (McCoy et al., 2007); and papaya BARWIN-like protein (CARWIN, PDB ID: 4JP7) as search model after modification using Chainsaw (Stein, 2008). The structure was refined using Phenix (Adams et al., 2011) and COOT (Emsley and Cowtan, 2004) was used for model building into σa-weighted 2Fo-Fc and Fo-Fc electron density maps. R and Rfree were monitored to evaluate the validity of the refinement protocol, and the stereochemistry of the model was assessed using Molprobity (Chen et al., 2010). The coordinates and structure factors have been deposited in Protein Data Bank (PDB ID: 7KSN).

The data-collection, processing and refinement statistics are shown in Table 1.

**TABLE 1:** Data-collection, processing and refinement statistics.

	**SUGARWIN2**
Detector	Pilatus3 6M
Cell parameters (Å) a, b, c, α, β, γ	227.51, 227.51, 227.51, 90.0, 90.0, 90.0
Space group	*F*4132
Resolution (Å)	52.20–1.51 (1.55–1.55)
X ray source	DLS I24
λ (Å)	0.96863
Multiplicity	75.7 (72.2)
R_pim_ (%)	1.7 (42.1)
CC (1/2)	0.999 (0.791)
Completeness (%)	100.0 (100.0)
Reflections	5,976,527 (415,898)
Unique reflections	78,971 (5,764)
*I*/σ	23.2 (2.4)
Reflections used for refinement	78,910
R (%)	16.26
R_free_ (%)	18.50
N° of protein atoms	1,848
N° of ligant atoms	0
B (Å^2^)	13.88
Coordinate error (Å)	0.13
Phase error (°)	16.52
**Ramachandran plot**	
Favored (%)	96.72
Allowed (%)	3.28
Outliers (%)	0.0
All-atom clashscore	4.23
**RMSD from ideal geometry**	
r.m.s. bond lengths (Å)	0.014
r.m.s. bond angles (°)	1.291
*PDB ID*	7KSN

### Gene Sequences and Phylogenetic Analysis

Protein sequences homologous to SUGARWINs 1 and 2 were searched for in 106 Embryophyta genomes obtained from the NCBI RefSeq Genome database and in the genomes of *Saccharum spontaneum* (Zhang et al., 2018) and the Brazilian hybrid cultivar *Saccharum spp.* SP80-3280 (Souza et al., 2019), using the BLASTp algorithm (Altschul et al., 1990). For genes with multiple isoforms of splicing, only the longest one was retrieved for further analyses. Protein domains were predicted using the InterProScan software (Jones et al., 2014).

A Maximum Likelihood phylogenetic tree inference was predicted using the IQ-TREE software v.1.6.12 (Nguyen et al., 2015) employing the automatic amino acid substitution model selection. Branch support values were calculated using 100 bootstrap replications. The protein sequence alignment used in the phylogenetic tree analysis was predicted using MAFFT software v7.453 (Katoh and Standley, 2013) and filtered using trimAL v.1.4 with the -automated1 method (Capella-Gutierrez et al., 2009).

### Molecular Modeling, Molecular Dynamics and Homology Modeling of SUGARWINs

All the SUGARWIN systems were prepared in AmberTool19 (Olsson et al., 2011) using the ff99SB force field for proteins and TIP3P for water molecules. The conversion of the original structures to the amber topology and coordinates, as well as the addition of water molecules and counter ions was performed with the *tleap* program. A buffer of at least 10 Å between protein and the periodic box wall was used. For the SUGARWIN2 model, the input coordinates were taken from the crystallographic structure presented in this work (PDB ID: 7KSN). For SUGARWIN1, SUGARWIN3 and SUGARWIN4 the coordinates were taken from those obtained from comparative models. Protonation states of the protein were assigned at pH 7.5 using the PROPKA3 (Olsson et al., 2011) web server.

The MD simulations were performed using NaMD, version 2.14 (Olsson et al., 2011). The time step and the temperature were set to 2.0 fs and 300 K, respectively. Simulations were performed in the isothermal-isobaric ensemble (NPT) and periodic boundary conditions were employed, with Van der Waals interactions computed using a 12.0 Å cut-off, and electrostatic contributions calculated *via* a particle mesh Ewald method using a grid with 1.0 Å spacing. Prior to MD, each of the systems was energy minimized with a steepest-descent energy algorithm. Altogether, MD simulations were performed for a duration of 100 ns after equilibration, with snapshots of the system being saved every 20 ps, giving 5,000 snapshots for subsequent analysis. All the trajectories were analyzed in Bio3D package (Grant et al., 2006).

The homology modeling was performed using Modeller v9.24 (Marti-Renom et al., 2000) employing the coordinates of the Sugarwin2 as model. 200 models were built for each SUGARWIN and the best model was selected by using internal Modeller energy score.

## Results

### PR-4 Phylogenetic Proposition

A total of 436 PR-4 protein sequences were identified in our searches of Embryophyta genomes. These sequences were present in 101 out of 106 analyzed genomes, corresponding to 95.3% of the total. PR-4 genes were found to be absent from the five species of Asterales (3) and Lamiales (2), but were present in the form of multiple copies in 94 of the remaining genomes (Supplementary Table 1).

**FIGURE 1:**
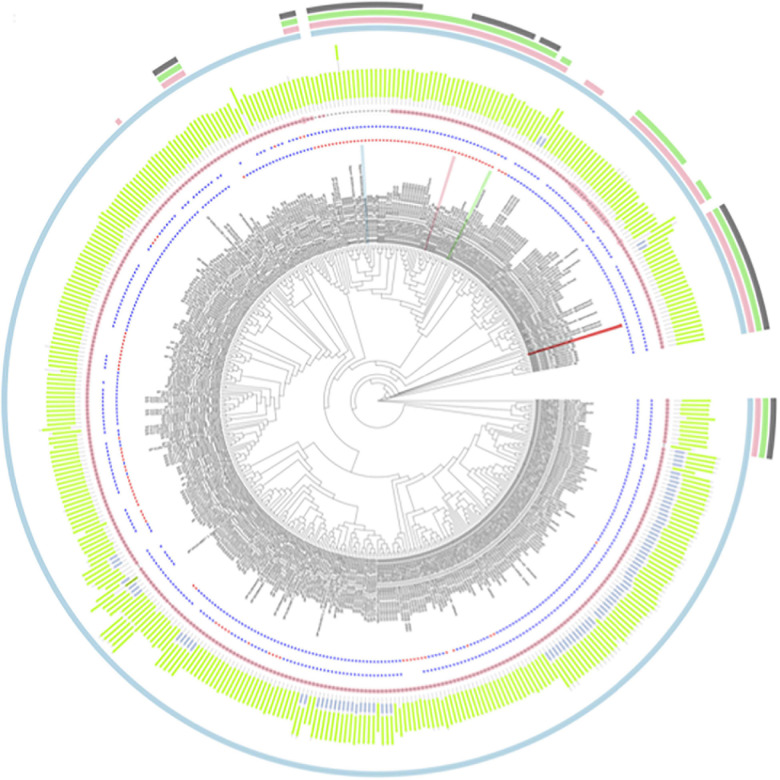
Phylogenetic analysis for 436 PR-4 proteins found in the genome annotation from 106 Embryophyta species. Labels colored as red, blue, green and pink highlight the positions of SUGARWINS 1, 2, 3, and 4, respectively. The first- and second-star rings represent the amino acid composition in the positions H11 and H111, respectively, with blue and red stars representing histidine and asparagine residues, respectively. The pink dot plots represent the number of additional residues in relation to SUGARWIN2 in the loop corresponding to the SUGARWIN1 variable loop, the larger the circle, the greater the number of additional amino acids. In the next circle, green and blue bars represent the positions of the Barwin and chitin recognition domains, respectively, according to PFAM results. The set of four outermost circles shows the sequences from the taxonomic groups: Magnoliopsida (light blue), Liliopsida (pink), Poales (light green), and PACMAD (gray).

The BARWIN domain (Pfam ID: PF00967) was found in all protein sequences analyzed, whereas a “chitin recognition protein” domain (Pfam ID: PF00187) was found in 92, which corresponds to 21.1%. In addition, the chitin recognition domain was absent in all sequences from Liliopsida species. Among the 436 PR-4 protein sequences found in our searches, 312 and 101 of them had histidine or asparagine at position 11 [H42—SUGARWIN1; N37—SUGARWIN2; N43—SUGARWIN3; N38—SUGARWIN4], respectively, whereas 362 and 18 had histidine or asparagine at position 113 [H142—SUGARWIN1; H135—SUGARWIN2; N142—SUGARWIN3; H137—SUGARWIN4] (Figure 1). These two positions have been associated with the different catalytic activities attributed to PR-4 proteins and a total of 268 of the sequences examined (61.5%) had both histidine residues H11 and H113 in their catalytic site.

### Crystallography and Molecular Modeling

PR-4 proteins lacking the His11-His113 pair are less common and do not have its crystal structure, therefore, we solved the crystal structure of SUGARWIN2. Analysis of the protein structure shows an asparagine residue at position 11, which is followed by a loop shorter by two residues when compared either with SUGARWIN1, either with the canonical structures of BARWIN and CARWIN.

**FIGURE 2:**
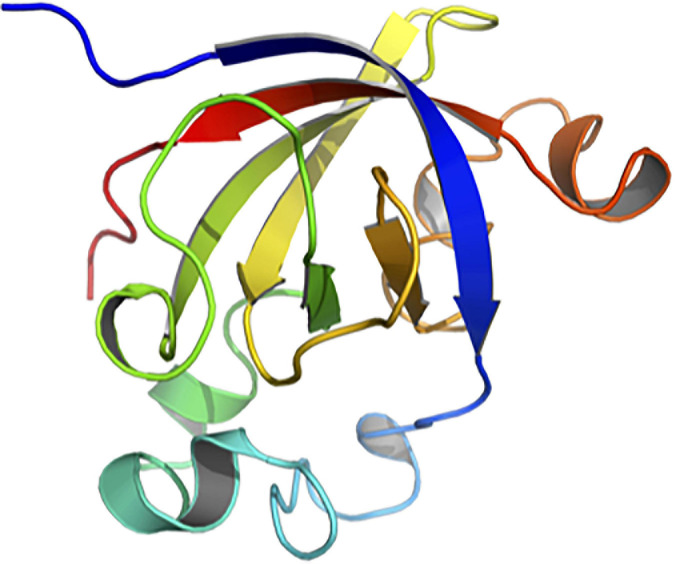
Crystallographic structure of SUGARWIN2. The structure of SUGARWIN2 at 1.55 Å shown in cartoon-style using the “Chainbow” representation in which the N-terminus is dark blue and the C-terminus red. SUGARWIN2 has the classical BARWIN-like fold. The variable loop between the first and second β-strands (blue and green respectively) is two residues shorter than in previously reported structures.

**FIGURE 3:**
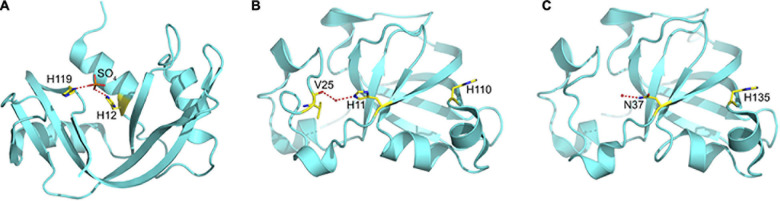
The Catalytic Site in **(A)** Ribonusease A, **(B)** CARWIN, and **(C)** SUGARWIN2. In RNaseA, the catalytic histidines are orientated toward one another and bound to a sulfate ion which mimics the substrate where hydrolytic attack occurs. In CARWIN, although present, the histidines are orientated away from one another and the side chain of His11 is connected to the insertion within the variable loop by water mediated hydrogen bonds. In order to cleave RNA, it is expected that RNase active enzymes (including SUGARWIN1) must undergo a large conformational change to bring the two histidines into the correct orientation for catalysis. The presence of Asn37 in SUGARWIN2, in place of the histidine, makes this protein RNase inactive.

SUGARWIN2 crystallizes in cubic space group F4_1_32 with two molecules per asymmetric unit. The structure was refined to R and R_free_ values of 16.26 and 18.50% respectively. The two molecules are in the same conformation with an RMSd of 0.18 Å on Cα positions. When compared with CARWIN from papaya, the RMSd is 0.45 Å, indicating a very well conserved 3D fold. SUGARWIN2 presents the classical BARWIN fold (CATH classification 2.40.40) consisting of a six-stranded β-barrel surrounded by 4 α-helices and several loops (Figures 2, 3). Three disulfide bonds (Cys30-Cys62, Cys51-Cys85, Cys65-Cys122) help to maintain the BARWIN domain stability.

Classical ribonucleases, such as RNase A, possess two catalytic histidine residues in their active sites. These residues are involved in a general acid-base mechanism leading to substrate hydrolysis (Figure 3A). The absence of RNase activity in SUGARWIN2 is believed to be due to the substitution of His11 by an asparagine. SUGARWIN1, BARWIN and CARWIN proteins have both histidines at the same positions and display ribonuclease activity. In both RNase A (PDB ID: 5RSA) and CARWIN (PDB ID: 4JP7) the distance between Cα’s of the two histidines is about 10 Å. This is also the case in the inactive SUGARWIN2 structure reported here, albeit that one has been substituted by an asparagine residue. However, different from RNase A, where the histidine sidechains face one to another in a catalytically competent arrangement, CARWIN structure shows that these residues point to opposite directions. Similar observation is reported for the histidine/asparagine pair in SUGARWIN2. Taking all together, we suggest that SUGARWIN1 and other ribonuclease PR-4 proteins must provide a significant flexibility at the active site to assure the catalytic histidine properties for catalysis (see below). In addition, the histidine sidechains face one another in a catalytically competent arrangement, whereas in CARWIN they point in opposite directions and the same is true of the histidine/asparagine pair in SUGARWIN2 (Figure 3). Together, these observations suggest that in the case of SUGARWIN1 and other ribonuclease active PR-4 proteins, there must be significant flexibility to the active site region, in particular the catalytic histidines, to re-orientate into positions appropriate for catalysis (see below).

According to our observations there is a variable loop following the first β-strand which is two residues shorter in SUGARWIN2 when compared with the ribonuclease-active SUGARWIN1, BARWIN and CARWIN. In the latter, the inserted residues are Lys-Val which form a type I β-turn, where the valine carbonyl is hydrogen bonded to the catalytic His11 *via* a water molecule, thereby connecting the active site to the variable loop. The equivalent insertion in SUGARWIN1 is Asn-Ala and would be expected to behave similarly. In addition, for the SUGARWIN2 the shorter loop forms a type II β-turn (facilitated by the presence of Gly24) and there is no connectivity to the asparagine, which replaces His11 at the active site. Instead, the asparagine side chain appears not to be fixed in a unique conformation but rather, adopts different rotamers in the two monomers of the asymmetric unit. However, in both of them it is structurally disconnected from the variable loop which lies further away due to the lack of the two-residue insertion.

**FIGURE 4:**
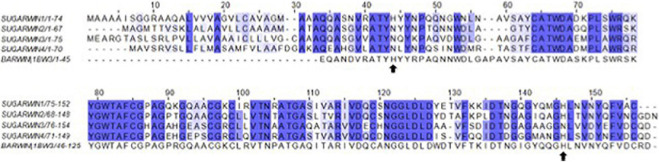
Alignment of SUGARWINs and BARWIN. The alignment (ClustalW) of the full sequences, including the signal peptide, highlights a histidine at alignment position 44 (His11) in BARWIN and SUGARWIN1. SUGARWINs 2, 3 and 4 show the replacement of histidine by asparagine at alignment position 44.

**FIGURE 5:**
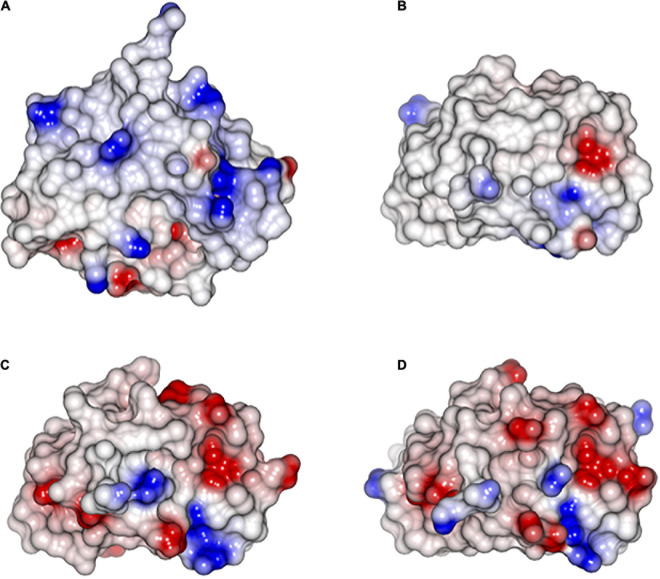
Electrostatic surface of the SUGARWINs. **(A)** SUGARWIN1; **(B)** SUGARWIN2; **(C)** SUGARWIN3; **(D)** SUGARWIN4. SUGARWIN1 is more –positive (blue) than the remaining SUGARWINs. The electrostatic surface analysis shows a mainly electronegative surface in SUGARWIN 2, 3 and 4 suggestive of poor binding to RNA, if any.

SUGARWIN1 and SUGARWIN2 sequence analysis reveals several substitutions affecting charged amino acid residues. The majority of these alterations correspond to the substitution of basic residues in SUGARWIN1 by neutral residues in SUGARWIN2 (Figure 4). These changes have a dramatic consequence for the pI of the two proteins: 8.78 for SUGARWIN1 and 4.82 for SUGARWIN2. By generating a homology model for SUGARWIN1 based on the crystal structure of SUGARWIN2 it was possible to map these residues onto the two molecular surfaces. Figure 5 shows that they give rise to significant differences to the surface electrostatic potential. Such differences would be expected to have consequences for the binding partners of the two proteins and the molecular recognition processes which govern their interaction. For example, the binding of RNA would be expected to be facilitated in the case of SUGARWIN1 compared with SUGARWIN2 based on electrostatic considerations. These changes, together with the absence of the catalytic histidine (His11), help to explain the lack of RNase activity of the latter.

Molecular modeling was also used to build 3D structures for these recently described SUGARWINs and the electrostatic surface analysis shows a mainly negative surface predicting a weak binding to RNA (Figure 4). Molecular modeling electrostatic surface analysis were also used to build 3D structures for the newly-identified SUGARWIN3 and SUGARWIN4. Our data shows a mainly negative surface predicting a weak binding to RNA (Figure 4). Future research is necessary to functionally characterize these SUGARWINs, however, we suggest that the lack of a complete RNase active site and a less positively charged molecular surface may go hand-in-hand.

### Molecular Dynamics

The observation that SUGARWIN2 has a two residues deletion in the variable loop, which is connected by hydrogen bonds to the active site His11 in active RNases, stimulated us to investigate the dynamics of the molecule as a whole. This was further motivated by the observation, that reorientation of the catalytic residues would be expected to occur during catalysis. In order to analyze the consequences of this deletion on structural mobility, a molecular dynamics approach was employed, starting from the crystal structure of SUGARWIN2. Molecular modeling of the remaining 3 SUGARWINs (SUGARWIN1, SUGARWIN3 and SUGARWIN4) was also performed.

**FIGURE 6:**
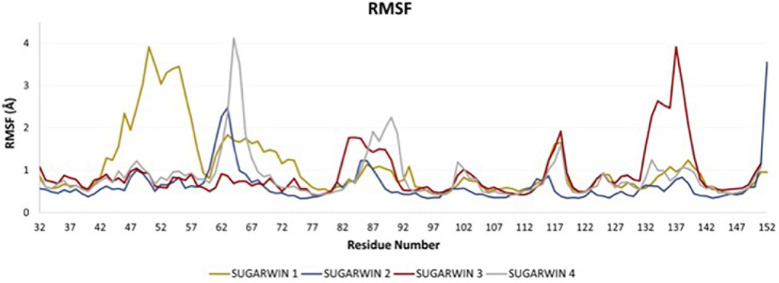
RMSF of the SUGARWINs. Root mean square fluctuation (RMSF) analysis for SUGARWINs 1, 2, 3 and 4 as derived from molecular dynamics simulations. Residues are numbered in reference to SUGARWIN1 complete sequence.

The molecular dynamics of the crystal structure of SUGARWIN2 together with the derived models for SUGARWINs 1, 3 and 4 shows a very similar pattern of intrinsic movements in all cases. The most notable features of this analysis are the large peaks associated with the variable loop in the model for SUGARWIN1 (residues 42–61) which are absent from the remaining structures. The RMSF data from the molecular dynamics simulations (Figure 6) highlight the mobility of the variable loop, as shows a major peak for SUGARWIN1 along residues 44–58. The loop begins at residue H11 [42—SUGARWIN1] which is believed to be involved in RNase activity and which, in the structure of CARWIN (H11), is coupled to an insertion within the loop itself by water mediated hydrogen bonds. SUGARWIN2 was the least flexible structure found in this analysis, with a minor peak between residues 60–65. SUGARWIN3 showed a major peak between residues 132 and 140, a region which precedes the second catalytic residue assumed to be essential for RNA hydrolysis (His113). SUGARWIN3 is the only structure to show this mobility and is also unique in having an asparagine at this position. Together with the behavior observed for SUGARWIN1 this suggests that structural mobility close to the catalytically implicated residues, H11 and H113, may be correlated with the type of amino acid which occupies these positions. SUGARWIN4 shows a single major peak involving residues 62–68, whereas other less prominent peaks are observed in all structures.

**FIGURE 7:**
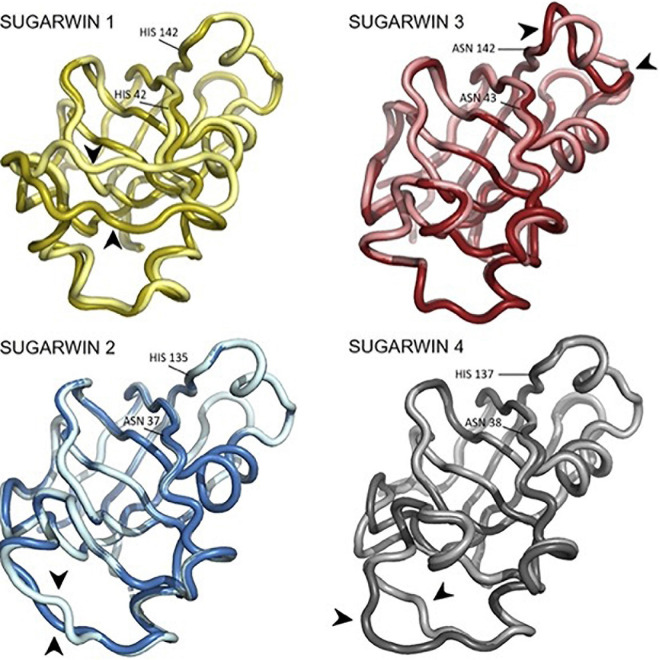
PCA analysis of the SUGARWINs. Structures of SUGARWINs 1, 2, 3 and 4 generated by Bio3D PCA analysis indicating the main movements of each protein. Brighter and darker colors indicate extreme points of movements, extracted from PCA generated frames. Black arrowheads highlight regions of major flexibility. Residues in equivalent positions to the RNase active site histidines are indicated.

**FIGURE 8:**
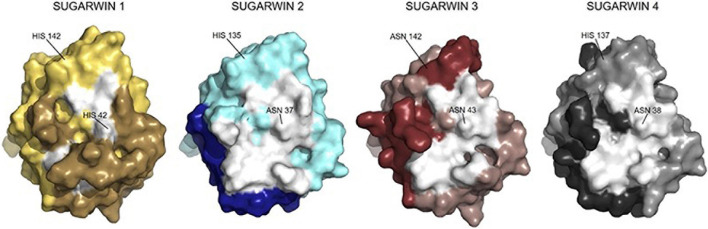
Structures of SUGARWINs with emphasis on the chitosan binding site. Structures of SUGARWINs 1, 2, 3 and 4 in surface representation showing, in white, the regions previously identified as the binding site for chitosanase activity in SUGARWIN2. Darker colors indicate regions of major flexibility according to the RMSF analysis. These superpose with the binding site regions in a few cases. Residues in equivalent positions to the RNase active site histidines are indicated.

PCA analysis for each simulation was performed in order to identify putative sequence movements, which were then grouped and graphically represented. This data allowed the observation of the most flexible regions of each structure which correspond to the most representative group for each simulation (PC1), and are shown in Figure 7. Regarding the active site of the chitosanase function, only SUGARWIN1 showed significant movement in this region due to the flexibility of the variable loop described above (Figure 8). The mobility of this region may therefore be related to the ability for SUGARWIN1 to act as both a chitosanase and a ribonuclease, whereby different catalytic activities would be related to different loop conformations. We suggest that it is the dynamic nature of SUGARWIN1 (particularly the variable loop), which allows functional diversity implying that different structural conformations are a necessary requirement for different catalytic activities (Figure 8).

## Discussion

Plant response to biotic stresses such as herbivore insects and fungal pathogens involves a wide set of complex interactions, which cause profound physiological, chemical and morphological adaptations (Silva-Filho and Vivanco, 2017). Previous studies using transcriptomic data showed only two genes encoding for PR-4 proteins in sugarcane: SUGARWIN1 and SUGARWIN2 (Medeiros et al., 2012; Franco et al., 2014, 2019). However, in this work, we expand this observation by two additional protein sequences with distinct structural characteristics, named SUGARWIN3 and SUGARWIN4.

SUGARWIN1 exhibits RNase, chitinase and chitosanase activity, whereas SUGARWIN2 has only chitosanase activity (Franco et al., 2019). SUGARWIN3 and SUGARWIN4 have yet to be functionally characterized. Therefore, in order to shed light on the known differences among this protein family, we determined the three-dimensional structure of SUGARWIN2, which was crystallized and diffracted to 1.55 Å. Although SUGARWIN2 does not have RNase activity, its crystal structure shows the classic BARWIN fold (a six stranded β-sheet surrounded by four α-helices and several loops). In addition, we were able to build homology models for the remaining SUGARWINs for structural comparisons.

Examination of the structures for the four variants revealed that SUGARWIN1 presented at least three unique properties. Firstly, it is the only SUGARWIN to possess the catalytic residue, His11, associated with RNase activity; secondly, it is the most flexible of the four structures and finally, it has the most positive electrostatic surface potential. We argue that this unique combination of features renders SUGARWIN1 an active ribonuclease.

Several studies have correlated the RNase activity of BARWIN with the presence of two highly conserved histidine residues: at positions 11 and 113 (in relation to the BARWIN mature protein) [CARWIN (H11 and H110), WHEATWIN (H11 and H113) and SUGARWINs as referred above] (Caporale et al., 2004; Bertini et al., 2009; Huet et al., 2013; Franco et al., 2019). In addition, Bertini et al. (2009) also evaluated the relationship between the ribonuclease activity of SUGARWIN counterparts and their antifungal properties. It has been demonstrated that two different mutations to His11 (H11G and H11L) of WHEATWIN partially inhibited RNase activity, pointing out the importance of this residue for catalysis. Furthermore, these authors also shown that the presence of the catalytic residues (His11 and His113, in WHEATWIN1) is also fundamental for the antifungal activity, at least in WHEATWIN1, thereby correlating the two phenomena. Huet et al. (2013) also reported that the two homologous histidine residues are important for RNase activity in CARWIN.

Although SUGARWINs 1, 2 and 4 all have His146, only SUGARWIN1 possesses both histidine residues. Franco et al. (2019) demonstrated the loss of SUGARWIN1’s RNase activity by mutating His11 to asparagine (H42N—SUGARWIN1). This strongly suggests that RNA hydrolysis by SUGARWIN1 occurs according to the classic acid-base mechanism involving both histidines, as seen in RNase A, RNase T1, BARWIN, CARWIN and WHEATWINs (Silva-Filho and Vivanco, 2017).

Although SUGARWIN1 has the catalytic machinery necessary for the hydrolysis of RNA, the SUGARWIN2 structure, similar to CARWIN’s, shows that the His residues at positions 11 and 113 (11 and 111—CARWIN; 37 and 135—SUGARWIN2) point to opposite directions. In the case of SUGARWIN1 the two catalytic histidines would be expected to be oriented similarly, as observed in our homology-based model. However, this arrangement is incompatible with the acid-base mechanism, which suggests that SUGARWIN1 must have sufficient intrinsic flexibility in order to reposition the catalytic diad in such a way as to be competent for catalysis. This hypothesis is compatible with the separation observed between the Cα atoms of Asn11 and His113 in SUGARWIN2 (10.6 Å), which is similar to that observed in bovine RNase A (8.9 Å), suggesting a putative rearrangement of the protein structure. For this reason, we investigated the dynamics of all four SUGARWIN structures with particular interest in SUGARWIN1.

SUGARWIN1 displayed significant flexibility throughout the entire region of the variable loop (residues 44–56), but particularly in its N-terminal portion (residues 44–51). This loop starts at the catalytic His11, which, as mentioned above, is only present in SUGARWIN1. Furthermore, this loop includes a two-residue insertion in SUGARWIN1 and this insertion is coupled to His11 *via* water mediated hydrogen bonds (Figure 3). It seems reasonable to assume that the altered dynamic behavior of the variable loop in SUGARWIN1 is due to these notable structural differences. A direct association between loop flexibility with RNase function has already been suggested, whereby the loop may be important for the binding and correct positioning of RNA in the active site as well as for RNA release after catalysis (Doucet et al., 2009; Menezes et al., 2014; Li and Hammes-Schiffer, 2019).

In contrast, SUGARWINs 2 and 4 showed significant structural mobility only within the C-terminal portion of the variable loop (residues 60–68, Figure 6), whilst SUGARWIN3 showed a markedly different profile altogether with a peak toward the C-terminus (residues 132–141), which precedes the latter catalytic histidine. It is worth emphasizing that SUGARWIN3, which is unique in presenting increased mobility in this region, is the only SUGARWIN that has an asparagine residue in place of histidine at this position (H142N—SUGARWIN3). Overall, it would therefore appear that differences in chain mobility would be related to the nature of the amino acids occupying the catalytic positions 11 and 113 as well as to the variable loop insertion in the case of SUGARWIN1. These results are in agreement with previous data (Ludvigsen and Poulsen, 1992; Bertini et al., 2009, 2012; Huet et al., 2013; Franco et al., 2014, 2019).

All known PR-4 ribonucleases, such as BARWIN, CARWIN and WHEATWIN, possess the two-residue insertion in the variable loop (Doucet et al., 2009; Menezes et al., 2014; Li and Hammes-Schiffer, 2019). Movement of this loop may be critical to give His11 the necessary structural liberty to move from the position seen in our model for SUGARWIN1 (and CARWIN) in order to form the RNase active site. This seems particularly evident on examining the CARWIN structure in which one of the two inserted residues of the loop holds the histidine sidechain to its mainchain carbonyl *via* a water-mediated hydrogen bond. The additional flexibility associated with the variable loop in SUGARWIN1 may be sufficient to decouple His11 from the loop insertion, thereby allowing it to adopt an alternative conformation—one compatible with RNase hydrolysis. However, we should point out that during our simulations we did not observe His11 pointing toward His113 as might be expected. This may suggest that the generation of a mature RNase active site may depend not only on the flexibility of the variable loop but also on association with the substrate itself, RNA, through an induced-fit like mechanism.

Evidence for the reorientation of His11 comes from the NMR structure of BARWIN, where it is partially rotated in the direction of His113, its partner in the RNase active site. This is facilitated by the variable loop adopting a different conformation in BARWIN, such that His11 is not coupled to the insertion within the variable loop. This is a direct evidence that changes to the conformation of the variable loop could be related to liberating His11 in order to allow the formation of a mature RNase active site. Amongst the four different variants only SUGARWIN1 has the histidine at position 11 and the necessary structural plasticity.

For the ribonuclease activity of SUGARWIN1 to occur it is not sufficient to have the catalytic machinery and the expected flexibility allowing the formation of the active site with the histidines residues properly oriented. In addition to these two features, it is also necessary for the enzyme to attract and bind the highly negatively charged substrate, RNA. Examination of the electrostatic potential of the four SUGARWINs revealed that SUGARWIN1 is, by far, the most basic protein. This is probably due to a series of amino acid substitutions leading to an accumulation of lysines and arginines residues on the molecular surface (Figure 3) and a significant increase in the pI in comparison to the remaining SUGARWINs. This alteration to the electrostatic properties of the SUGARWIN1 surface would be expected to back the formation of the enzyme-substrate complex thereby permitting RNA hydrolysis to occur.

Different from the RNase activity, both SUGARWIN1 and SUGARWIN2 are known to be active chitosanases. However, the major movement to the variable loop in SUGARWIN1 leads to a significant occlusion of the known chitosan binding site (Figure 8). Therefore, the loop movement which we consider to be critical for the RNase active site, simultaneously covers the chitosan binding site. This suggests that SUGARWIN1 (and by analogy BARWIN, CARWIN and WHEATWIN) would be unable to simultaneously perform both catalytic functions. If the variable loop is positioned to expose the chitosan-binding site, then the RNase active site is not formed, as implied by the structure for SUGARWIN2. On the other hand, it seems likely that when the latter is correctly formed, then the chitosan-binging site will be occluded by the loop. Therefore, we propose that SUGARWIN1 might be considered as an enzyme with two different catalytic functions which are mutually exclusive.

No function has been described yet for the newly-described SUGARWINs (SUGARWIN3 and 4). It seems clear that no nuclease function might be associated with these proteins, since both lack the catalytic histidine at position 11. Additionally, SUGARWIN3 also has an asparagine residue replacing the histidine at position 113. Nevertheless, according to the structural analyses, it might be expected that both proteins display chitosanase activities. This is consistent with the fact that it is only in SUGARWIN1 that we observe the variable loop crosses over the chitosan binding site. For the other isoforms, the binding site for the chitosan substrate remains exposed during the simulations. We have previously shown that for SUGARWIN1 both the RNase and chitinase activities do not impair its chitosanase function. As mentioned above this is probably associated with the structural flexibility in the region of the variable loop which is most notable in SUGARWIN1, the only variant with a known nuclease function (SUGARWIN1).

Our phylogenetic analysis showed that both histidines related to RNase activity and a variable loop of the same size to that found in the variable region of SUGARWIN1 are present in the majority of the Embryophyta PR-4 protein sequences and are widespread in the phylogenetic tree. In contrast, proteins with an asparagine residue at position 11 were found only in a few genomes and do not form a separate clade. These results show that both histidine and the flexible loop observed in SUGARWIN1 may have been present in the PR-4 protein from the Embryophyta ancestral species and that independent replacements by asparagine may have happened in the evolutionary history of this group. One of these replacements may have occurred in the common ancestor of Poales originating the sequence that gave rise to SUGARWIN2, SUGARWIN3 and SUGARWIN4 (Figure 1).

## Conclusion

Thus far only two PR-4 proteins have had their tridimensional structures solved: BARWIN from Barley [PDB ID: 1BW3 (Ludvigsen and Poulsen, 1992)] and CARWIN from papaya [PDB ID: 4JP6 (Huet et al., 2013)], both of which contain H11 and H113 (H11 and H110 in CARWIN) and with chitinase and RNase activity. Here, we described the first tridimensional structure of a PR-4 protein with no RNase activity and with the H44N replacement: SUGARWIN2. Comparison of the structures, dynamics and surface properties of the two proteins allows us to propose that (1) the more positive surface potential of SUGARWIN1 aids in binding the substrate RNA, (2) flexibility of the variable loop is essential for the formation of the RNase binding site which otherwise would have the catalytic histidines pointing to opposite directions as observed in the crystal structures, and (3) the flexibility of the loop also occludes the chitosan binding site suggesting that the two catalytic activities are mutually exclusive. Unexpectedly, despite its lack of RNAse activity, SUGARWIN2 seems to be the more relevant for sugarcane defense against pathogenic fungi, since its expression is strongly upregulated after 10 days of *C. falcatum* treatment, whereas SUGARWIN1 showed a moderate increase in gene expression under the same conditions. This may imply that a more specialized enzyme is required for this purpose. We also describe here two new SUGARWIN proteins, SUGARWIN3 and SUGARWIN4, which are predicted, based on the above analyses, to lack RNase activity. Further studies are being conducted to better understand these new proteins. The exact details of the catalytic capabilities of each of these enzymes appears to be a complex function of several factors including the residues which comprise the active site, the intrinsic flexibility of the structures themselves and the surface electrostatics necessary for substrate recognition.

## Data Availability Statement

The datasets presented in this study can be found in online repositories. The names of the repository/repositories and accession number(s) can be found in the article/Supplementary Material.

## Author Contributions

MS-F: design of the experiments, lab infrastructure, providing research funds, discussions, and writing. All authors contributed to the article and approved the submitted version.

## Conflict of Interest

The authors declare that the research was conducted in the absence of any commercial or financial relationships that could be construed as a potential conflict of interest.

## Publisher’s Note

All claims expressed in this article are solely those of the authors and do not necessarily represent those of their affiliated organizations, or those of the publisher, the editors and the reviewers. Any product that may be evaluated in this article, or claim that may be made by its manufacturer, is not guaranteed or endorsed by the publisher.
