# Supplementary material for: Structural and Evolutionary Analyses of PR-4 SUGARWINs Points to a Different Pattern of Protein Function
Source: Front Plant Sci. 2021 Sep 9;12:734248. doi: 10.3389/fpls.2021.734248 (PMC8458871; doi:10.3389/fpls.2021.734248)
Supplement: Supplementary file 1 [file Data_Sheet_1.pdf]

Supplementary Material

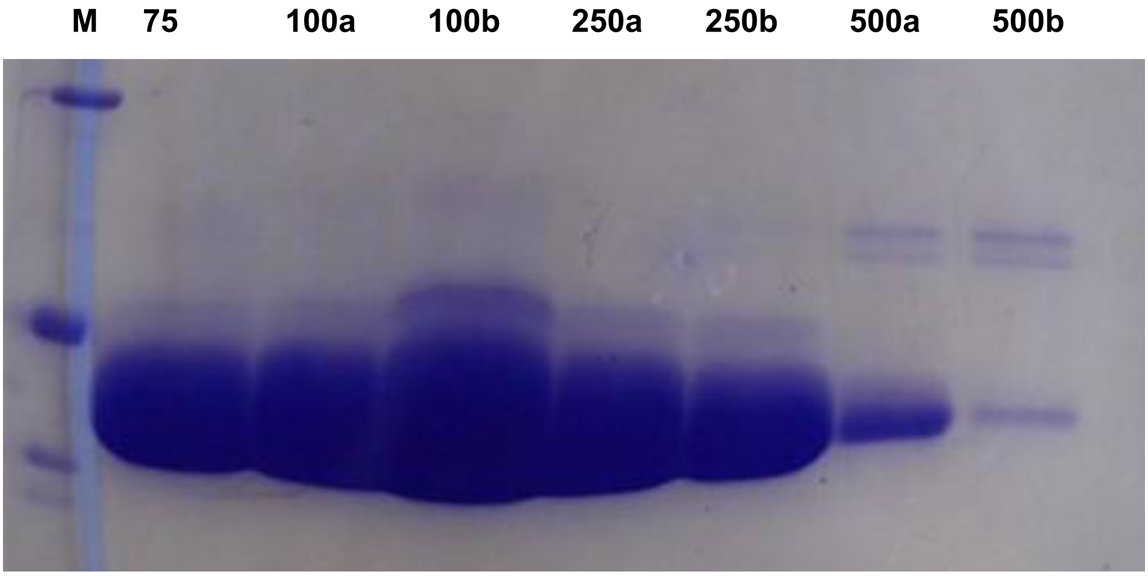

Supplementary Figure 1. 12% SDS-PAGE showing the purification ofSUGARWIN2 by IMAC. Lane M Molecular Weight Marker; Lane 1-7 aliquots of fractions eluted with crescent concentrations of imidazole (75 to 500 mM). The arrow indicates the band representing purified SUGARWIN2.

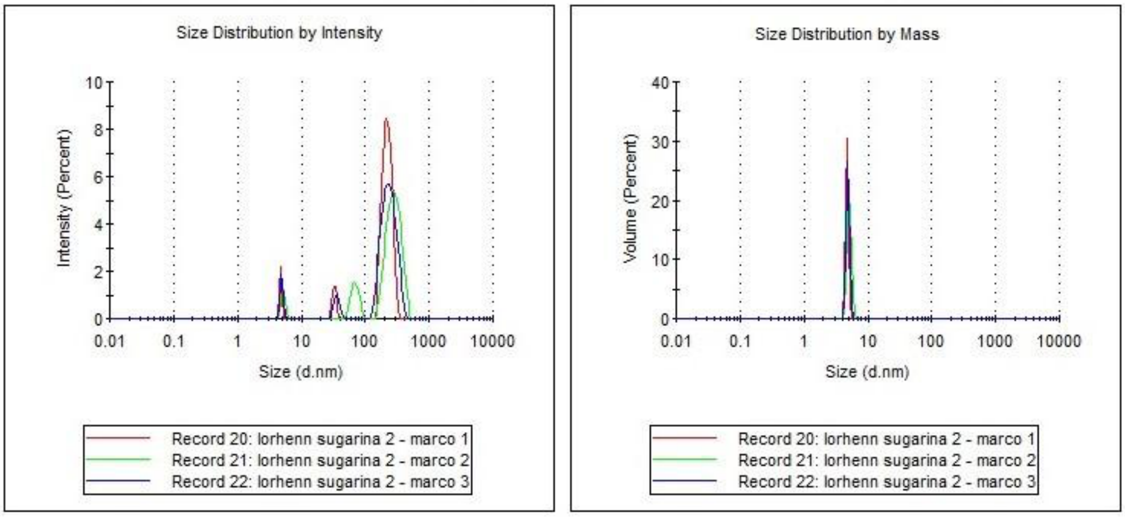

Supplementary Figura 2. DLS of SUGARWIN2. The DLS of SUGARWIN2 showed a monodisperse peak that corresponded to 99.7% of the mass consistent with the mass of the protein.

Supplementary Table 1. Distribution of sequences among the clades of the phylogenetic tree

					Clades			
					**C1**	**C2**	**C3**	**C4**
**Sequences with H or N residues in position 11**								
					Percentage (%)			
				**H11**	15	97	6	100
				**N11**	75	3	94	0
**Number of genes**								
Subclass	Clade	Order	Clade	Species				
Petrosaviidae		Asparag.		*Asparagus officinalis* (Aoff)	1	0	0	0
				Dendrobium catenatum (Dcat)	0	1	0	0
				Phalaenopsis equestris (Pequ)	0	1	0	0
	Commelinids	Arec.		*Elaeis guineensis* (Egui)	2	0	0	0
				Phoenix dactylifera (Pdac)	2	0	0	0
		Zingib.		*Ensete ventricosum* (Even)	1	0	0	0
				Musa acuminata subsp. malaccensis (Macu)	3	0	0	0
				Musa balbisiana (Mbal)	2	0	0	0
		Poales		*Ananas comosus* (Acom)	2	0	0	0
				Carex littledalei (Clit)	1	0	0	0
			BOP	*Aegilops tauschii subsp. Tauschii* (Atau)	3	3	1	0
				Triticum dicoccoides (Tdic)	6	6	2	0
				Triticum turgidum subsp. durum (Ttur)	1	6	0	0
				Brachypodium distachyon (Adis)	1	1	0	0
				Oryza brachyantha (Obra)	0	2	0	1
				Oryza meyeriana var. granulata (Omey)	0	2	0	1
				Oryza sativa Japonica Group (Osat)	0	3	0	1
			PACMAD	*Eragrostis curvula* (Ecur)	3	1	2	6
				Dichanthelium oligosanthes (Doli)	2	0	0	2
				Panicum hallii (Phal)	2	0	1	2
				Setaria italica (Sita)	3	0	1	2
				Setaria viridis (Svir)	3	0	1	2
				Sorghum bicolor (Sbic)	1	0	1	2

				Zea mays (Zmay)	1	0	2	2
				Miscanthus lutarioriparius (Mlut)	3	0	4	9
				Saccharum spp. (SP80-3280)	8	0	14*	18**
				Saccharum spontaneum (Sspo)	1	0	2	5
		Al.		*Zostera marina* (Zmar)	0	0	2	0

*Includes SUGARWIN2

**includes SUGARWIN1.

***Al., Alismatales; Zigib., Zingiberales; Arec., Arecales; and Asparag., Asparagales.
